# Machine learning-based approaches for identifying human blood cells harboring CRISPR-mediated fetal chromatin domain ablations

**DOI:** 10.1038/s41598-022-05575-3

**Published:** 2022-01-27

**Authors:** Yi Li, Shadi Zaheri, Khai Nguyen, Li Liu, Fatemeh Hassanipour, Betty S. Pace, Leonidas Bleris

**Affiliations:** 1 Bioengineering Department, The University of Texas at Dallas, Richardson, TX USA; 2 Center for Systems Biology, The University of Texas at Dallas, Richardson, TX USA; 3 Department of Mechanical Engineering, The University of Texas at Dallas, Richardson, TX USA; 4 Department of Biological Sciences, University of Texas at Dallas, Richardson, TX USA; 5 Department of Pediatrics, Augusta University, Augusta, GA USA

**Keywords:** Genetic engineering, Data processing, Machine learning

## Abstract

Two common hemoglobinopathies, sickle cell disease (SCD) and β-thalassemia, arise from genetic mutations within the β-globin gene. In this work, we identified a 500-bp motif (Fetal Chromatin Domain, FCD) upstream of human ϒ-globin locus and showed that the removal of this motif using CRISPR technology reactivates the expression of ϒ-globin. Next, we present two different cell morphology-based machine learning approaches that can be used identify human blood cells (KU-812) that harbor CRISPR-mediated FCD genetic modifications. Three candidate models from the first approach, which uses multilayer perceptron algorithm (MLP 20-26, MLP26-18, and MLP 30-26) and flow cytometry-derived cellular data, yielded 0.83 precision, 0.80 recall, 0.82 accuracy, and 0.90 area under the ROC (receiver operating characteristic) curve when predicting the edited cells. In comparison, the candidate model from the second approach, which uses deep learning (T2D5) and DIC microscopy-derived imaging data, performed with less accuracy (0.80) and ROC AUC (0.87). We envision that equivalent machine learning-based models can complement currently available genotyping protocols for specific genetic modifications which result in morphological changes in human cells.

## Introduction

The Clustered Regularly Interspaced Short Palindromic Repeats (CRISPR) genome editing technology, which is adapted from an immune system analog found in archaea and prokaryotes, has been applied to exceedingly broad scientific, industrial, and medical domains at an exceptional pace since the first demonstration in cells^1–8^. One particularly exciting domain is CRISPR-based therapeutics, which as of today, has found applications in several areas: blood disorders, diagnostics and therapeutics in cancer, eye diseases, chronic infections, neurodegenerative disorders, and protein-folding disorders^9^.

Two most common hemoglobinopathies, sickle cell disease (SCD) and β-thalassemia, arise from genetic mutations within the β-globin gene. These mutations result in deficient or absent β-globin synthesis, which in turn lead to oxygen being disassociated from the hemoglobin and eventually result to conformational changes in red blood cells^10,11^. There is no cure available for these disorders except bone marrow transplantation (BMT) when a suitable donor is available, and most treatments are mainly aimed at relieving symptoms and preventing complications. Recently, the CRISPR technology has been used to reactivate the expression of fetal hemoglobin, which can take the place of defective adult hemoglobin, and has shown remarkable results in improving the quality of life in patients^12^.

Machine learning, which can yield models for pattern recognition, classification, and prediction from acquired data, has been widely used in biological studies ranging from protein folding prediction^13^ to cancer prognosis^14^. There are two main types of machine learning methods: (1) supervised learning (e.g. random forest, support vector machine), which derive the relationship between a set of input variables (features) and a designated dependent variable (label) from training instances and subsequently can be used to predict on new instances, and (2) unsupervised learning (e.g. clustering), which infer patterns from data without known labels^15^. More recently, deep learning, a collection of new machine learning techniques extended from classical neural networks, has gained popularity due to its better performance compared to existing best-in-class machine learning algorithms across several fields including linguistics^16^, high-energy physics^17^, computational chemistry^18^, and biology^19^.

One area that has received particular attention in recent years is the classification of different cell types (e.g. different blood cell types)^20–23^, states (apoptotic and healthy cells)^24–27^, and genotypes^28^. In one study^29^, Suzuki and colleagues developed a convolutional neural network (CNN) that was at least 90% accurate in classifying whole blood cells, peripheral blood mononuclear cells, human colon cancer cells, and human T lymphoma cells, using imaging flow. Similarly, in our previous work^24^, we have shown that machine learning can be an efficient and cost-effective approach in identifying live and apoptotic human cells. Suzuki and colleagues also demonstrated that, using label-free, brightfield (BF) microscopy images, machine learning models (logistical regression) can be used to predict cells harboring ubiquitin–proteasome system-related genetic mutations with good performance (ROC AUC = 0.773)^28^.

This work is motivated by our recent discovery of a 500-bp motif upstream of human ϒ-globin locus (named as Fetal Chromatin Domain, FCD, Supplementary Materials/FCD sequence). We show that the removal of this motif in human blood cells (i.e., KU-812) reactivates the expression of fetal hemoglobin. Herein, we explore cell morphology-based machine learning approaches to classify KU-812 cells with or without the genetic modifications within the FCD domain.

## Materials and methods

### Cell culture

The KU-812 parental and derived cells were acquired from the American Type Culture Collection (ATCC, catalog number: CRL-1573) and maintained at 37 °C, 100% humidity and 5% CO_2_. The cells were grown in Dulbecco’s modified Eagle’s medium (DMEM, Invitrogen, catalog number: 11965–1181) supplemented with 10% Fetal Bovine Serum (FBS, Invitrogen, catalog number: 26140), 0.1 mM MEM non-essential amino acids (Invitrogen, catalog number: 11140–050), and 0.045 units/mL of Penicillin and 0.045 units/mL of Streptomycin (Penicillin–Streptomycin liquid, Invitrogen, catalog number: 15140). To pass the cells, the confluent cell culture was diluted in fresh medium at a ratio of 1:10. When applicable, 2 µg/mL puromycin (ThermoFisher Scientific, catalog number: A1113803) was added to the growth medium.

### Generation of FCD-HT monoclonal stable cell line

To generate the FCD-HT monoclonal stable cell line, approximately 10 million human KU-812 cells were seeded onto a 10 cm petri dish. 16 h later, the cells were transiently transfected with 4.5 μg of PCMV-SpCas9-U6-sgRNA-L, 4.5 μg of PCMV-SpCas9-U6-sgRNA-R, and 1 μg of the donor plasmid using the JetPEI reagent (Polyplus Transfection). 48 h later, puromycin was added at the final concentration of 2 μg/mL. The selection lasted 2 weeks, after which the surviving clones were pooled to generate the polyclonal stable cells. Next, to remove the puromycin resistance gene-T2A-mKate cassette, approximately 10 million of the polyclonal stable cells were seeded onto a 10 cm petri dish, and after 16 h were transfected with 10 μg of EF1-Flpase (unpublished data) using the JetPEI reagent. 48 h later, single cells were isolated using flow cytometry. The established monoclonal stable cell line was confirmed to be heterozygous by genotyping and further expanded and maintained in the complete growth medium.

### Genotyping of FCD-HT monoclonal stable cell line

The genomic DNAs were isolated from FCD-HT monoclonal stable cells using DNeasy Blood&Tissue Kit (Qiagen). The transcripts containing the CRISPR-targeting region was amplified with primers P13 and P14. The PCR products were then subjected to gel electrophoresis and Sanger sequencing using primers P13 and P14.

### Quantitative reverse transcription-PCR (qRT-PCR)

For measurement of mRNA levels of various human globin variants, total RNA was extracted using the RNeasy Mini Kit (Qiagen, #74104). First strand synthesis was performed using the QuantiTect Reverse Transcription Kit (Qiagen, #205311). Quantitative PCR was performed using the KAPA SYBR FAST Universal qPCR Kit (KAPABiosystems, #KK4601), with GAPDH levels used for normalization. Quantitative analysis was performed using the 2^−ΔΔCt^ method. Fold-change values are reported as mean with standard deviation. Primers used for ϒ-globin were (P15) 5′-GGCAACCTGTCCTCTGCCTC-3′ and (P16) 5′-TAGGAAGCCATTTCTGCCTTG-3′. Primers used for GAPDH were (P17) 5′-AATCCCATCACCATCTTCCA-3′ and (P18) 5′-TGGACTCCACGACGTACTCA-3′.

### Flow cytometry

FCD-WT, FCD-HT, HCT116 and PUF3.1 HCT116 cells from a 10-cm petri dish were washed with 5 mL PBS buffer, and subsequently trypsinized with 2 mL 0.25% Trypsin–EDTA at 37 °C for 5 min. Trypsin–EDTA was then neutralized by adding 10 mL of complete medium. The cell suspension was centrifuged at 1000 rpm for 5 min and after removal of supernatants, the cell pellets were re-suspended in 5 mL PBS buffer. The cells were analyzed on a BD Reforest flow analyzer. The voltages (V) for each channel were: 270 for FSC-A, 270 for FSC-H, 270 for FSC-W, 280 for SSC-A, 280 for SSC-H, and 280 for SSC-W.

### Differential interference contrast (DIC) microscopy

Approximately 50,000 FCD-WT or FCD-HT cells were seeded on 12-well plates (Greiner Bio-One) in the complete medium. Cells were imaged using an Olympus IX81 microscope in a Precision Control environmental chamber. The images were captured using a Hamamatsu ORCA-03 Cooled monochrome digital camera. The filter set was Differential Interference Contrast (DIC) with magnification at 40×. After obtaining the images, Adobe Photoshop was used to isolate individual cells with fixed size at 100 pixels by 100 pixels.

### Machine learning model training and testing

For flow cytometry-derived dataset, a Dell desktop computer (Intel Core i7-10700 CPU @ 2.90 GHz, Windows 10 enterprise 64-bit OS and 32 GB RAM) was used to conduct the machine learning modeling. Scikit-Learn, a free Python machine learning library, was used to conduct all model training and testing procedures. For DIC microscopy-derived dataset, a Lenovo Laptop (Intel Core i7-10510 CPU @ 1.80 GHz, Ubuntu 20.04 OS and 16 GB RAM) was used to conduct the deep learning modeling. The Keras library in TensorFlow was used to conduct all model training and testing procedures. Other Python libraries, including NumPy, Pandas, and Matplotlib, were also included for data analysis and presentation.

### Performance metrics

Performance of different models was evaluated using threshold dependent and independent metrics, which include:

1. precision: this parameter measures how accurate a model is when predicting cells being at live state.
  Precision = TP/(TP + FP), where TP refers to correctly predicted live cells and FP refers to falsely predicted live cells.
2. recall: this parameter measures the model’s ability to correctly predict live cells from actual live cells.
  Recall = TP/(TP + FN), where TP refers to correctly predicted live cells and FN refers to falsely predicted apoptotic cells.
3. true positive rate (TPR): this parameter measures the model’s ability to correctly predict live cells from actual live cells.
  TPR = TP/(TP + FN), where TP refers to correctly predicted live cells and FN refers to falsely predicted apoptotic cells.
4. false-positive rate (FPR): this parameter measures the model’s level of falsely predicting live cells from actual apoptotic cells.
  FPR = FP/(FP + TN), where FP refers to falsely predicted live cells and TN refers to correctly predicted apoptotic cells.
5. accuracy: this parameter determines the success of correctly predict live and apoptotic cells from overall data.
  Accuracy = (TP + TN)/(TP + FP + TN + FN), where TP refers to correctly predicted live cells, FP refers to falsely predicted live cells, FN refers to falsely predicted apoptotic cells, and TN refers to correctly predicted apoptotic cells.

## Results

### Generation and characterization of heterozygous FCD-deficient KU-812 cell model (FCD-HT)

**Figure 1:**
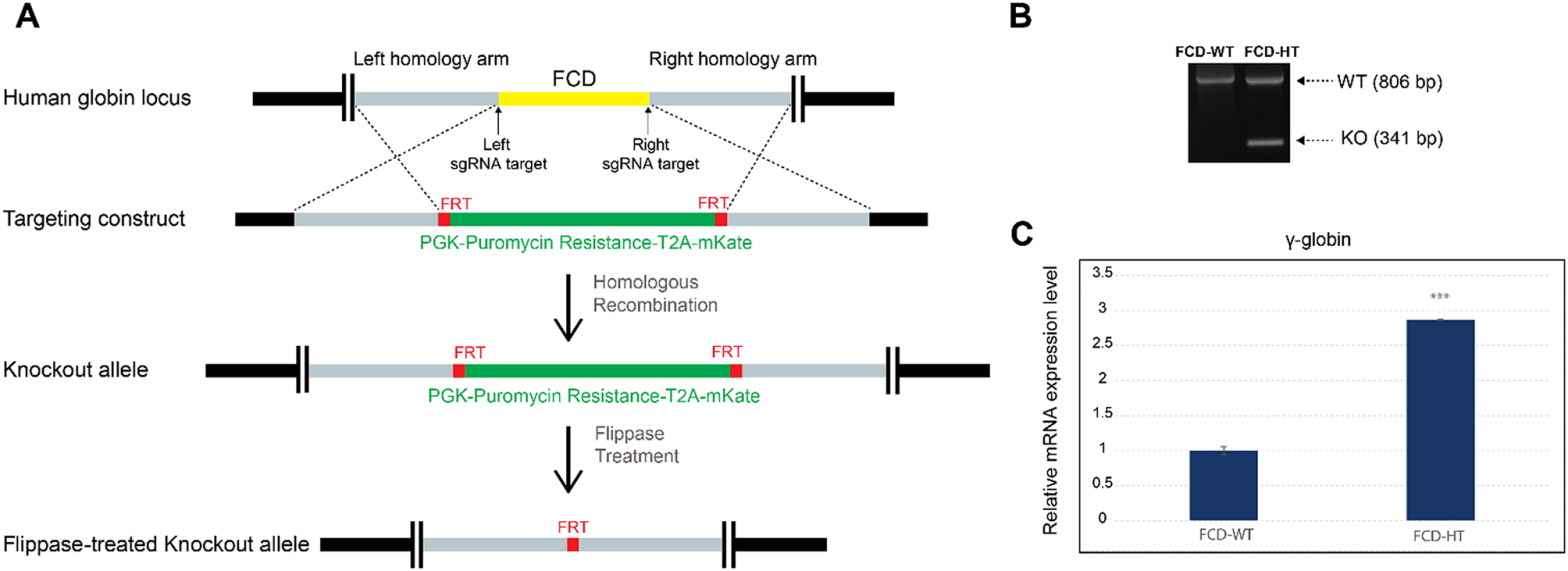
Generation and characterization of heterozygous FCD-deficient KU-812 cell model (FCD-HT). (**A**) Schematic illustration of the CRISPR/SpCas9 homologous recombination process to remove the FCD motif within human globin locus. The polyclonal stable cell line was established using 2 weeks of puromycin selection (2 µg/mL). Subsequently, the puromycin resistance gene transcript, which was flanked by flippase recognition target (FRT) sites, was removed using flippase. Finally, monoclonal stable cells were established using FACS single cell sorting. (**B**) Genotyping of FCD-HT monoclonal stable cell. Genomic DNAs were harvested from FCD-WT and FCD-HT cells and the transcript containing the FCD motif or FRT site was PCR amplified and subsequently subjected to gel electrophoresis. The stable cell line yielded two bands corresponding to both the wild type (806 bp) and FCD-knockout (341 bp) alleles, confirming its heterozygous status. (**C**) Quantitative reverse transcription-PCR (qRT-PCR) assay showed that compared to FCD-WT cells, the mRNA level of ϒ-globin significantly increased in FCD-HT cells (2.87-fold). *** denotes p-value < 0.001.

The CRISPR/SpCas9 technology was used to generate the FCD-deficient model in KU-812 cells, which were established from the peripheral blood of a patient with chronic myelogenous leukemia^30^. Briefly, the parental cells were transiently transfected with CRISPR/SpCas9 complex which targets both left and right genomic regions flanking the FCD motif, along with a homologous recombination repair template containing a puromycin resistance gene transcript (Fig. 1A). The polyclonal stable cell line was then established after 2 weeks of puromycin selection (2 µg/mL). Subsequently, to avoid potential interference with the transcriptional activities of the globin genes, the puromycin resistance gene transcript (~ 2.2 kb), which was flanked by flippase recognition target (FRT) sites, was removed using flippase. Finally, monoclonal stable cells were established using FACS single cell sorting.

To characterize the stable monoclonal cell line, genomic DNA was isolated using DNeasy Blood&Tissue Kit (Qiagen), and subsequently the transcript containing the FCD motif or FRT site was amplified using primers P13 and P14 (Supplementary Table 1). The PCR products were then subjected to gel electrophoresis and as shown in Fig. 1B and Supplementary Fig. 1A, the monoclonal cell line yielded two distinct bands corresponding to both the wild type (806 bp) and FCD-knockout (341 bp) alleles, confirming its heterozygous status (named as FCD-HT). Both bands were further extracted and subjected to Sanger sequencing, which confirmed that the FCD sequence was successfully removed in the FCD-Knockout allele (Supplementary Fig. 1B and Supplementary Materials/Sanger_FCD_Knockout_Allele.seq). To determine how the FCD-removal affects the ϒ-globin expression, total RNAs were extracted using the RNeasy Mini Kit from KU-812 and FCD-HT cells and the relative expression of ϒ-globin transcript was determined using quantitative reverse transcription-PCR (qRT-PCR). As shown in Fig. 1C, the mRNA level of ϒ-globin significantly increased in FCD-HT cells (2.87-fold compared to its parental KU-812, named as FCD-WT), which is consistent with our hypothesis that the removal of the FCD motif may reactivate the expression of fetal hemoglobin.

### Flow cytometry-based data collection and visualization for FCD-WT and FCD-HT cells

To prepare cell morphology-based predictive models differentiating FCD-WT, in which all ϒ-globin alleles contain the FCD motif, and FCD-HT cells, which contain both wild-type and FCD-knockout ϒ-globin alleles, we first used flow cytometry assay to record 6 features (FSC-A, FSC-H, FSC-W, SSC-A, SSC-H, and SSC-W). Specifically, FSC (forward scatter) measures the light scatter along the path of the laser (A: area, H: height, and W: width), and is dependent on the diameter/size of the cell. In contrast, SSC (side scatter) measures the light scatter perpendicular to the path of the laser (A: area, H: height, and W: width), and provides information about the internal complexity (granularity) of a cell. In total, 192,772 FCD-WT cells (labeled as 0) and 185,544 FCD-HT cells (labeled as 1) were included (the ratio of labels 0 and 1 = 1.04, Supplementary Table 2). Next, this initial dataset was randomly split into training and testing datasets at a ratio of 80:20 (size of training dataset: size of testing dataset). Specifically, the training dataset contains 302,652 cells (label 0: 154,180 cells, label 1: 148,472 cells, Supplementary Fig. 2 and Supplementary Table 3), and the testing dataset contains 75,664 cells (label 0: 38,592 cells, label 1: 37,072 cells, Supplementary Fig. 3 and Supplementary Table 4).

**Figure 2:**
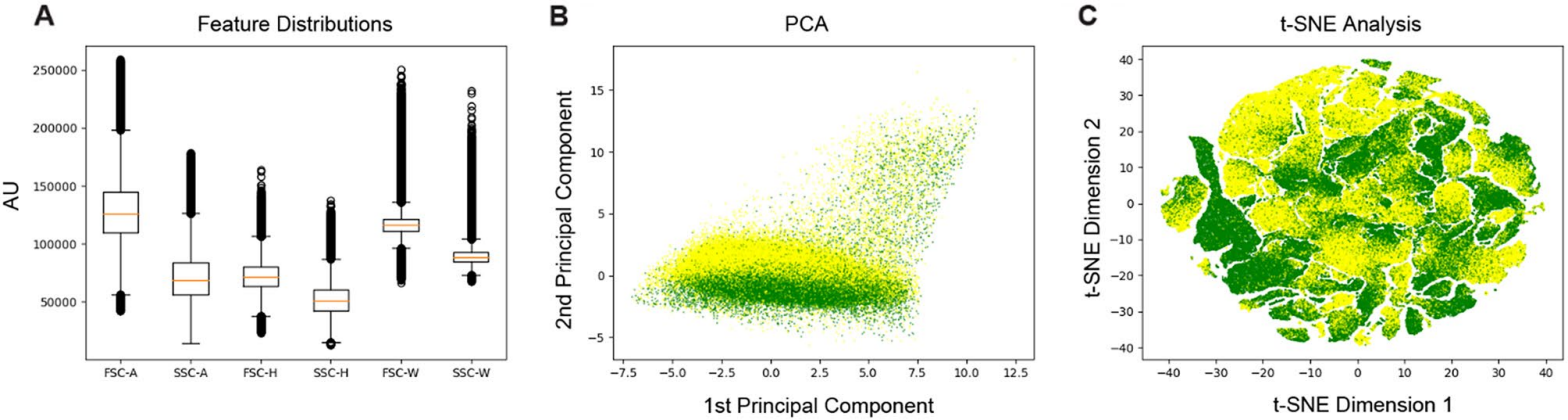
General statistics and visualization of the training dataset. (**A**) Box plot of the training dataset. The means of the six features (FSC-A, SSC-A, FSC-H, SSC-H, FSC-W, and SSC-W) varied significantly with the maximal ratio larger than 2.0-fold (mean_FSC-A_/mean_SSC-H_ = 2.47), indicating that standardization of the original training and testing datasets are required. (**B**) Visualization of the standardized training dataset using PCA (green: FCD-WT, yellow: FCD-HT). (**C**) Visualization of the standardized training dataset using t-SNE (green: FCD-WT, yellow: FCD-HT).

We first compared the absolute readings among the 6 features using box plotting. As shown in Fig. 2A, the means of these features varied significantly with the maximal ratio larger than 2.0-fold (mean_FSC-A_/mean_SSC-H_ = 2.47), indicating that standardization of the original training and testing datasets are required (standardized training and testing datasets in Supplementary Tables 5 and 6, respectively). Subsequently, the standardized training dataset was subjected to two dimensionality reduction algorithms, principal component analysis (PCA) and t-distributed stochastic neighbor embedding t-SNE (t-SNE). As shown in Fig. 2B (PCA) and Fig. 2C (t-SNE), the two cell subpopulations (FCD-WT: green, FCD-HT: yellow) overlapped significantly and were not linearly separable, which implies that non-linear modeling approaches such as multilayer perceptron may be required for classification purposes.

### Cell morphology-based machine learning models using flow cytometry-derived data

A general workflow as described in our previous study was adopted to build and test various cell morphology-based machine learning models using flow cytometry-derived data^24^. In total, five (5) supervised learning algorithms (logistical regression, random forest, k-nearest neighbor, support-vector machine, and multilayer perceptron) were included (model hyperparameters in Supplementary Table 7). Briefly, logistical regression is a statistical model which uses a logistic function to model a binary dependent variable. The model itself is non-linear but can be transformed into a linear regression via link functions. Random forest algorithm is an ensemble learning method which constructs “an ensemble” of decision trees at the training step. K-nearest neighbor algorithm for classification assumes that data points with same classification labels tend to be in proximity. Support-vector machine (SVM) relies on constructing one or a set of hyperplanes in high-dimensional space and remains one of the most robust supervised classification methods. Multilayer perceptron (MLP) belongs to the family of feedforward artificial neural network (ANN), and typically consist of three layers of nodes: an input layer, a hidden layer, and an output layer.

First, using tenfold cross-validation, we screened all models with the standardized training dataset, and adopted the filtering conditions as (1) the mean accuracy > 0.80, and (2) the standard deviation of accuracy < 0.10, which were derived from our previous study using the same 6 flow cytometry-based features to predict mammalian cell states^24^. In total, one (1) logistic regression model (Supplementary Table 8), 94 random forest models (Supplementary Table 9), 96 k-nearest neighbor models (Supplementary Table 10), two (2) SVM models with linear kernel (Supplementary Table 11), 25 SVM models with Gaussian kernel (Supplementary Table 12), and 893 MLP models (Supplementary Table 13) were selected.

Next, all selected models (1111) were trained using the training dataset, and subsequently applied to the standardized testing dataset and subjected to secondary filtering conditions as (1) precision when predicting FCD-HT cells > 0.80, and (2) recall when predicting FCD-HT cells > 0.80. As shown in Supplementary Table 14, only 533 MLP models survived this additional filter.

**Figure 3:**
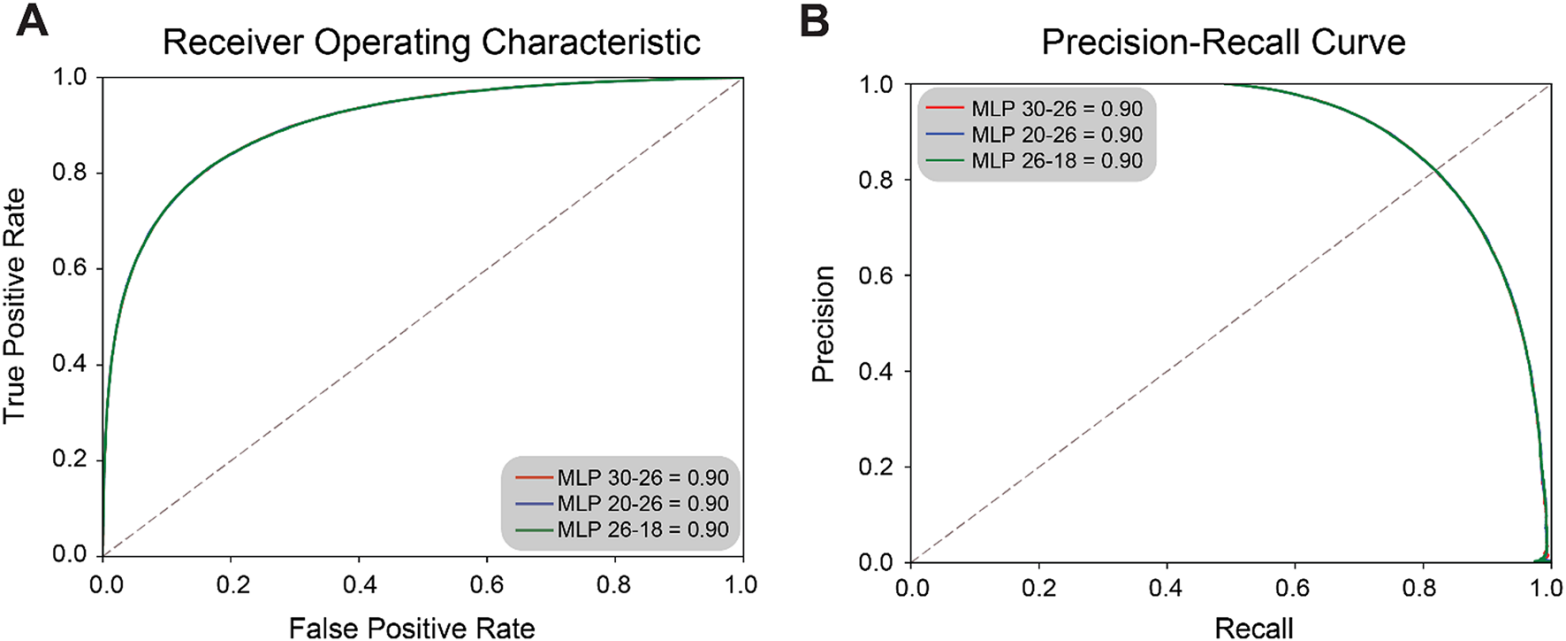
Predictive performances of the three candidate MLP models on FCD-HT cells. Both (**A**) ROC curve and (**B**) Precision-Recall curve showed that the three MLP models (MLP 20-26, MLP 26-18, and MLP 30-26) can predict the FCD-HT cells with relatively high precisions and recalls (precision: 0.83, recall: 0.80, accuracy: 0.82, and AUC: 0.90).

Finally, we chose three MLP models with largest AUC values (Table 1, MLP 20-26: number of nodes in the first layer: 20/number of nodes in the second layer: 26, MLP 26-18: number of nodes in the first layer: 26/number of nodes in the second layer: 18, and MLP 30-26: number of nodes in the first layer: 30/number of nodes in the second layer: 26), and plotted both the receiver operating characteristics (ROC, Fig. 3A) and precision-recall curves (Fig. 3B). The three models displayed essentially identical performance when predicting FCD-HT cells (precision: 0.83, recall: 0.80, accuracy: 0.82, and AUC: 0.90).

**Table 1:** Predictive performances of the three candidate learning models using flow cytometry-derived data.

Number of nodes in 1st layer	Number of nodes in 2nd layer	m00	m01	m10	m11	FCD-HT precision	FCD-HT recall	FCD-HT f-value	KU-812 precision	KU-812 recall	KU-812 f-value	accuracy	AUC
20	26	32,503	6089	7306	29,766	0.830177102	0.80292404	0.816323172	0.816473662	0.842221186	0.829147587	0.822967329	0.903213032
26	18	32,499	6093	7281	29,791	0.830202876	0.803598403	0.816684029	0.816968326	0.842117537	0.82935232	0.823244872	0.9031964
30	26	32,279	6313	7141	29,931	0.825819446	0.807374838	0.816492989	0.8188483	0.836416874	0.827539353	0.822187566	0.903233927

### Cell morphology-based machine learning models using microscopy-derived data

In addition to flow cytometry, cell morphology information can also be directly assessed using imaging^28^. Using a Differential Interference Contrast (DIC) microscopy, we prepared 1594 images of individual FCD-WT cells and 1695 images of FCD-HT cells (Supplementary Fig. 4), the ratio of FCD-WT and FCD-HT = 0.94). Next, this starting dataset was randomly split into the training and testing datasets at a ratio of 90:10 (size of training dataset: size of testing dataset). The final training dataset contains 2956 images (FCD-WT: 1433 images, FCD-HT: 1523 images), and the testing dataset contains 333 images (FCD-WT: 161 images, FCD-HT: 172 images).

Next, deep learning-based convolutional neural networks (CNNs) were used to construct genotype-predictive models. Two general CNN architectures were explored: (1) Type 1 (T1): (Conv-Conv-Pool)_n_, which was based on the VGG design^31^, and (2) Type 2 (T2): (Conv-Pool)_n_, which contained a single convolution layer for each repeat. For each type, different number of convolution numbers were tested (two, four and six layers for T1, and two, three, four, five layers for T2) until the final feature map reaches a dimension of zero. Since our image inputs have a relatively small size (100 pixels by 100 pixels), we fixed the filter size at 3 and when applicable, the Maxpooling pool size at 2. The detailed architectural designs were included in Supplementary Table 15.

**Figure 4:**
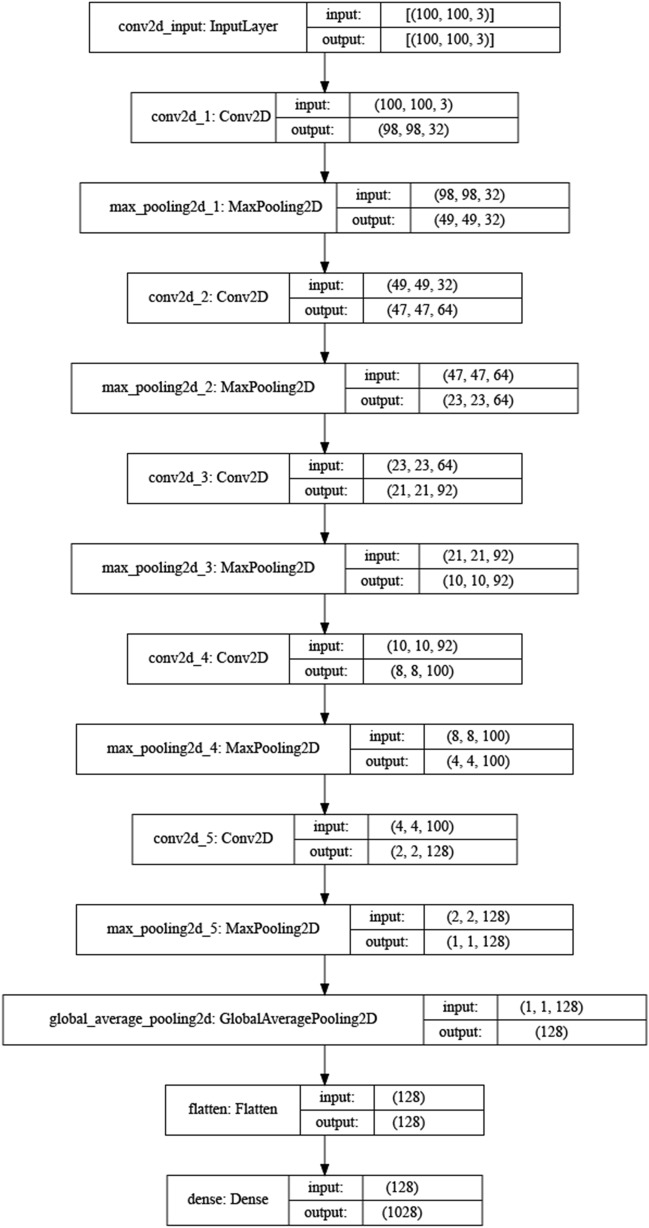
The T2D5 deep learning architecture. The model contained five convolutional layers (the numbers of each layer: 32, 64, 92, 100 and 128). Additionally, a Maxpooling layer was included after each convolution layer. Next, the outputs from convolutional layers were subjected to global average pooling and flattened for a fully connected layer (1028 nodes). Finally, a Softmax classifier, which applies a categorical cross-entropy loss function, was used.

As an example, for Type 2/5 layers (T2D5, Fig. 4), the numbers of layers at the feature extraction step were 32, 64, 92, 100 and 128 for each successive layer, and rectified linear unit (ReLU) was used as the activation function. Additionally, a Maxpooling layer was included after each convolution layer. Next, the outputs from convolutional layers were subjected to global average pooling and converted into a 1-dimensional vector (Flatten) for a fully connected layer (dense, 1028 nodes). Finally, a Softmax classifier, which applies a categorical cross-entropy loss function, was used, together with the adaptive moment estimation (ADAM) optimization algorithm.

First, all 7 candidate architectures were subjected to tenfold cross-validation using the training dataset. As shown in Supplementary Table 16, models from Type 2 showed better performance compared to those from Type 1. Specifically, the best-performing model of Type 2 (T2D5) showed a mean of accuracies from 10 cross-validation tests at 67.3% (Supplementary Fig. 5), while the best-performing model of Type 1 (T1D4) yielded a mean of accuracies at 58.3%.

**Figure 5:**
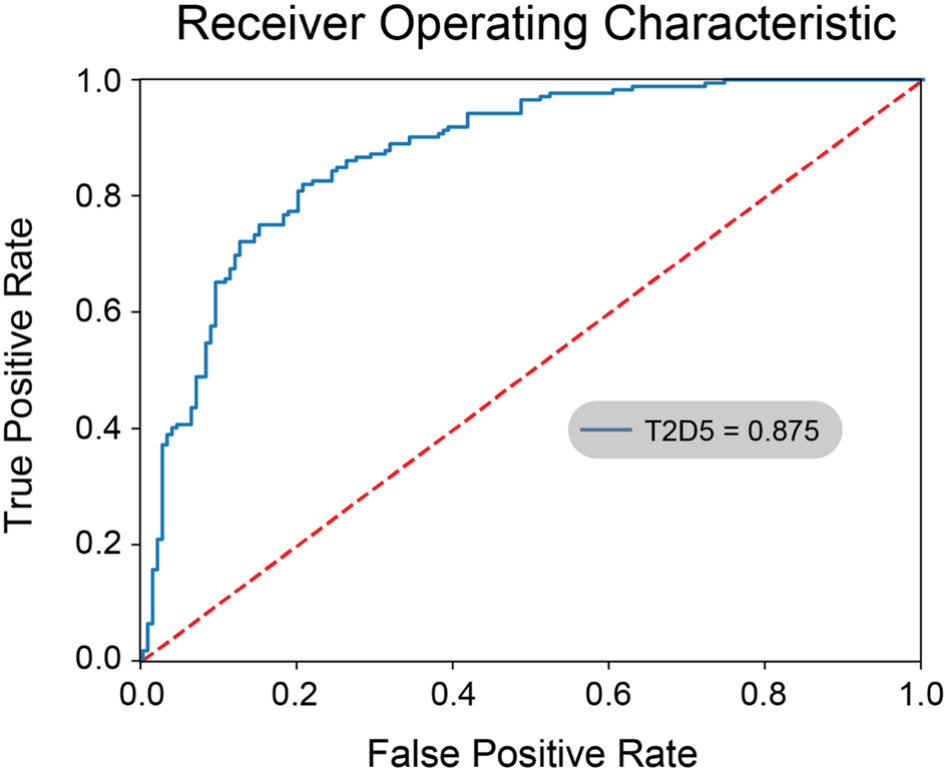
The ROC (Receiver Operating Characteristic) curve of the candidate deep learning model T2D5. The model showed relatively good performance when predicting FCD-HT cells (precision: 0.84, recall: 0.76, accuracy: 0.80, and AUC: 0.87).

We further trained models using all candidate architectures and the training dataset, and subsequently applied them to the testing dataset. As shown in Table 2, the architectures T2D5 displayed the best predictive outcome. Specifically, for FCD-HT cells, precision was 0.84, recall was 0.76, accuracy was 0.80 and AUC was 0.87 (Fig. 5).

**Table 2:** Predictive performances of the seven candidate learning models using microscopy-derived data.

Architecture	m00	m01	m10	m11	FCD-HT precision	FCD-HT recall	FCD-HT f-value	KU-812 precision	KU-812 recall	KU-812 f-value	Accuracy	AUC
Type 1 Depth 2 (T1D2)	79	82	68	104	0.559139785	0.604651163	0.581005587	0.537414966	0.49068323	0.512987013	0.54954955	0.553933
Type 1 Depth 4 (T1D4)	97	64	45	127	0.664921466	0.738372093	0.699724518	0.683098592	0.602484472	0.640264026	0.672672673	0.697783
Type 1 Depth 6 (T1D6)	76	85	94	78	0.478527607	0.453488372	0.465671642	0.447058824	0.472049689	0.459214502	0.462462462	0.500469
Type 2 Depth 2 (T2D2)	94	67	55	117	0.635869565	0.680232558	0.657303371	0.630872483	0.583850932	0.606451613	0.633633634	0.529684
Type 2 Depth 3 (T2D3)	103	58	53	119	0.672316384	0.691860465	0.681948424	0.66025641	0.639751553	0.649842271	0.666666667	0.642514
Type 2 Depth 4 (T2D4)	106	55	35	137	0.713541667	0.796511628	0.752747253	0.75177305	0.658385093	0.701986755	0.72972973	0.73653
Type 2 Depth 5 (T2D5)	136	25	42	130	0.838709677	0.755813953	0.795107034	0.764044944	0.844720497	0.802359882	0.798798799	0.874621

## Discussion

In this study, we have investigated two alternative approaches in predicting cell genotypes: (1) numeric data based on flow cytometry assay, and (2) imaging data based on DIC microscopy. Our analysis showed that the best performing models from approach 1 (MLP 20-26, MLP 26-18, and MLP 30-26) yielded better prediction results compared to the best model from approach 2 (T2D5) (Tables 1 and 2, ROC AUC values for MLP 20-26, MLP 26-18, and MLP 30-26: 0.90, for T2D5: 0.87). Multiple factors maybe impact the classification performance of the two approaches. For example, the resolution of our images was relatively low (100 pixels by 100 pixels). Additionally, compared to the training dataset from flow cytometry (302,652 cells), the size of imaging dataset was vastly smaller (3289 images). To overcome this challenge, we resorted to data augmentation techniques by applying zooming (range: 0.5–1.5x), rotation (range: 90°), width shifting (range: − 10 to 10 pixels), and height shifting (range: − 10 to 10 pixels) to the original training dataset (Supplementary Fig. 8)^32^. Together with original samples, the augmented training dataset contained 26,604 images (FCD-WT: 12,897 images, FCD-HT: 13,707 images), which was subsequently subjected to deep learning modeling using the T2D5 architecture. As shown in Supplementary Table 17, the newly acquired model did not yield better predictive performance. As an example, for FCD-HT cells, precision was 0.77, recall was 0.74, accuracy was 0.75, and ROC AUC was 0.75, which were lower than those of the original model (precision: 0.84, recall: 0.76, accuracy: 0.80, ROC AUC: 0.87). These results showed that synthetic samples do not always enhance the model performance in deep learning.

Lastly, for our default T2D5 model, while the accuracy approached 1.0 for the training dataset with the progression of epochs, the accuracy of the testing dataset plateaued at a much lower value (~ 0.8). This discrepancy indicated a potential overfitting (Supplementary Fig. 9). Consequently, we investigated additional tunings on overfitting/underfitting-controlling hyperparameters: L2 regularization (Ridge regression) weights (parameter: kernel_regularizer), and dropout values^33^. As shown in Supplementary Table 18, of the parameter space that we have scanned (L2 regularization weight: [0.000001, 0.00001, 0.0001, 0.01], dropout value: [0, 0.15, 0.2, 0.25, 0.3, 0.35, 0.4, 0.45]), none of the adjusted models (25) yielded better predictive performance compared to the original model (L2 regularization weight = 0, dropout value = 0), which indicated that additional factors may be involved in the divergency of accuracies between training and testing datasets.

It should be noted that data collected from our flow cytometry assay were based on fixed voltage values for each channel (“Materials and methods”/“Flow cytometry”, 270 for FSC-A, 270 for FSC-H, 270 for FSC-W, 280 for SSC-A, 280 for SSC-H, and 280 for SSC-W). In future studies, we will explore a wide range of voltage settings for all channels and systematically study how these modifications affect the modeling results. To compare the predictive performances between supervised and unsupervised learning algorithms, we additionally subjected our flow cytometry-derived dataset (the standardized training dataset) to two unsupervised clustering algorithms (k-means clustering and Gaussian mixture clustering) in parallel^34^. As shown in Supplementary Fig. 6 (SSC-A vs. FSC-A), the predicted distributive pattern for two subpopulations from k-means algorithm differed drastically from real genotype labels (Supplementary Fig. 2), which implied low accuracies on predicting FCD-WT and FCD-HT cells. Specifically, even with the best-performing labeling schema (green: FCD-WT, red: FCD-HT), the model yielded poor predictive performance when predicting FCD-HT cells (precision: 0.52, recall: 0.66, accuracy: 0.53). Similarly, as shown in Supplementary Fig. 7, the predictive model derived from Gaussian mixture clustering performed poorly, with precision at 0.61, recall at 0.25 and accuracy at 0.55 when predicting FCD-HT cells (green: FCD-WT, red: FCD-HT). It is interesting to note that in our previous study, which focused on predicting cell states using cell morphological information, supervised learning also performed significantly better than unsupervised learning. These results may be because compared to supervised learning, unsupervised learning uses less information (unknown outputs/labels), and thus may be less accurate when applied to data from complex systems such as human cells.

Although the MLP models provided better predictive performance compared to that of CNN, both methods could be useful for future studies. As shown in Supplementary Table 19, the instrumentation requirements for both approaches (regular flow cytometer and DIC microscope) are relatively affordable and typically available to university labs and research centers. The main advantages of our MLP approach include: (a) feasibility of collecting large number of training data (several millions in a typical experimental run), (b) relatively simple network architecture, and (c) in general faster convergency and shorter time required for the model training process. In contrast, the CNN approach may be more advantageous in (a) analyzing imaging-based dataset, which may contain more cell morphology information compared to the six FSC/SSC features extracted from our flow cytometry assay, (b) skipping feature identification/extraction step, which often requires deep domain knowledge, and (c) solving complicated classification problems due to its more extensive architectural design. Indeed, the latest flow cytometry platform, imaging flow cytometer^22^, can provide both FSC/SSC measurements and DIC images for each cell, which in theory allows the combination of both approaches.

It should be emphasized that since our training dataset contained only a specific cell type (i.e., KU-812) subjected to a specific CRISPR editing (CRISPR-mediated knockout of the FCD motif), the resulting models should not be used to differentiate any two cell types, or any cells with or without any CRISPR treatments. To demonstrate this scenario, we applied the candidate MLP 20-26 model to (1) a different human cancer cell type (HCT116, Supplementary Table 20), and (2) a human cancer cell line which was subjected to CRISPR-mediated knock-in (HCT116mut, Supplementary Table 21)^35^, both of which do not contain any mutations within the FCD motif (i.e., a correct model would classify both as FCD-WT genotype). Our results showed that the model yielded poor predictive performance on both cell lines (52.7% of HCT116 cells predicted as FCD-WT genotype, and 50.2% of HCT116mut as FCD-WT genotype), which confirmed that for each specific cell line and CRISPR editing event, the modeling process needs to be separately executed.

Typically, to establish and confirm a human stable cell line with desirable CRISPR-mediated genetic modifications, polyclonal cells need to be sorted into single cells, commonly on 96-well plates. Next, the single cells are allowed to propagate until sufficient genomic material can be extracted and subjected to PCR-based genotyping assays (Fig. 1). While the protocol is well established, the full pipeline can become time-consuming (up to several weeks for cell propagation step dependent on the cell proliferation rates) and labor-intensive (hundreds of monoclones may be needed for acquiring desirable genotypes). On the other hand, sophisticated imaging flow cytometry techniques, which record extensive physically measurable quantities (features) of the cells, have been used to identify cell subpopulations with specific traits (e.g. cell types, apoptotic states)^26,29,36,37^.

As an example, using next generation cell sorting and time-stretch imaging technologies, Jalali et al. achieved high accuracy (95%) when classifying OT-II white blood cells and SW-480 epithelial cells^38^. However, the required instrumentation (time-stretch imaging and customized microfluidic chip devices) may not be available to many research labs. In comparison, our flow cytometry and DIC microscopy-based machine learning approaches provide a unique balance between efficacy and availability, and theoretically can be applied to any engineered cells with genetic modifications known to introduce cell morphological changes.

In summary, we demonstrated the feasibility of using flow cytometry-based cellular information (FSC-A, FSC-H, FSC-W, SSC-A, SSC-H, and SSC-W) to predict specific cell genotypes using multilayer perceptron algorithms. Specifically, the three candidate MLP models, MLP 20-26, MLP 26-18, and MLP 30-26, achieved good predictive performance for predicting FCD-HT cells (AUC: 0.90). Additionally, we showed that deep learning framework (T2D5), when applied to DIC microscopy images, can also be indicative for certain genotyping purposes. We envision that both assays can be valuable and complementary to currently available genotyping protocols for engineered cell lines.

### Supplementary Information

Supplementary Table 1.

Supplementary Table 2.

Supplementary Table 3.

Supplementary Table 4.

Supplementary Table 5.

Supplementary Table 6.

Supplementary Table 7.

Supplementary Table 8.

Supplementary Table 9.

Supplementary Table 10.

Supplementary Table 11.

Supplementary Table 12.

Supplementary Table 13.

Supplementary Table 14.

Supplementary Table 15.

Supplementary Table 16.

Supplementary Table 17.

Supplementary Table 18.

Supplementary Table 19.

Supplementary Table 20.

Supplementary Table 21.

Supplementary Information 22.

Supplementary Information 23.

## References

[1] Wang H, Yang H, Shivalila CS (2013). One-step generation of mice carrying mutations in multiple genes by CRISPR/Cas-mediated genome engineering. Cell.

[2] Cong L, Ran FA, Cox D (2013). Multiplex genome engineering using CRISPR/Cas systems. Science.

[3] Hsu PD, Lander ES, Zhang F (2014). Development and applications of CRISPR-Cas9 for genome engineering. Cell.

[4] Ran FA, Cong L, Yan WX (2015). In vivo genome editing using *Staphylococcus aureus* Cas9. Nature.

[5] Jinek M, Chylinski K, Fonfara I, Hauer M, Doudna JA, Charpentier E (2012). A programmable dual-RNA-guided DNA endonuclease in adaptive bacterial immunity. Science.

[6] Mali P, Yang L, Esvelt KM (2013). RNA-guided human genome engineering via Cas9. Science.

[7] Moore R, Spinhirne A, Lai MJ (2015). CRISPR-based self-cleaving mechanism for controllable gene delivery in human cells. Nucleic Acids Res..

[8] Li Y, Nowak CM, Withers D, Pertsemlidis A, Bleris L (2018). CRISPR-based editing reveals edge-specific effects in biological networks. Cris J..

[9] Luthra R, Kaur S, Bhandari K (2021). Applications of CRISPR as a potential therapeutic. Life Sci..

[10] Asano H, Li XS, Stamatoyannopoulos G (1999). FKLF, a novel Krüppel-like factor that activates human embryonic and fetal β-like globin genes. Mol. Cell Biol..

[11] Li B, Ding L, Li W, Story MD, Pace BS (2012). Characterization of the transcriptome profiles related to globin gene switching during in vitro erythroid maturation. BMC Genomics.

[12] Frangoul H, Altshuler D, Cappellini MD (2021). CRISPR-Cas9 gene editing for sickle cell disease and β-thalassemia. N. Engl. J. Med..

[13] Senior AW, Evans R, Jumper J (2020). Improved protein structure prediction using potentials from deep learning. Nature.

[14] Kourou K, Exarchos TP, Exarchos KP, Karamouzis MV, Fotiadis DI (2015). Machine learning applications in cancer prognosis and prediction. Comput. Struct. Biotechnol. J..

[15] Giger ML (2018). Machine learning in medical imaging. J. Am. Coll. Radiol..

[16] Lakretz Y, Hupkes D, Vergallito A, Marelli M, Baroni M, Dehaene S (2021). Mechanisms for handling nested dependencies in neural-network language models and humans. Cognition.

[17] Azimi SA, Afarideh H, Chai JS, Kalinowski M, Gheddou A, Hofman R (2021). Classification of radioxenon spectra with deep learning algorithm. J. Environ. Radioact..

[18] Townshend RJL, Eismann S, Watkins AM (2021). Geometric deep learning of RNA structure. Science.

[19] Nabwire S, Suh HK, Kim MS, Baek I, Cho BK (2021). Review: Application of artificial intelligence in phenomics. Sensors..

[20] Habibzadeh Motlagh, M., Jannesari, M., Rezaei, Z., Totonchi, M. & Baharvand, H. Automatic white blood cell classification using pre-trained deep learning models: ResNet and inception, Vol. 10696, 1069612, (2018)10.1117/12.2311282.

[21] Huang X, Liu J, Yao J (2021). Deep-learning based label-free classification of activated and inactivated neutrophils for rapid immune state monitoring. Sensors.

[22] Nassar M, Doan M, Filby A (2019). Label-free identification of white blood cells using machine learning. Cytom. Part A..

[23] Lin Y-H, Liao KY-K, Sung K-B (2020). Automatic detection and characterization of quantitative phase images of thalassemic red blood cells using a mask region-based convolutional neural network. J. Biomed. Opt..

[24] Li Y, Nowak CM, Pham U, Nguyen K, Bleris L (2021). Cell morphology-based machine learning models for human cell state classification. npj Syst. Biol. Appl..

[25] Pischel D, Buchbinder JH, Sundmacher K, Lavrik IN, Flassig RJ (2018). A guide to automated apoptosis detection: How to make sense of imaging flow cytometry data. PLoS ONE.

[26] Feng J, Feng T, Yang C, Wang W, Sa Y, Feng Y (2018). Feasibility study of stain-free classification of cell apoptosis based on diffraction imaging flow cytometry and supervised machine learning techniques. Apoptosis.

[27] Vicar T, Raudenska M, Gumulec J, Masarik M (2019). Detection and characterization of apoptotic and necrotic cell death by time-lapse quantitative phase image analysis. bioRxiv..

[28] Suzuki G, Saito Y, Seki M (2021). Machine learning approach for discrimination of genotypes based on bright-field cellular images. npj Syst. Biol. Appl..

[29] Suzuki Y, Kobayashi K, Wakisaka Y (2019). Label-free chemical imaging flow cytometry by high-speed multicolor stimulated Raman scattering. Proc. Natl. Acad. Sci. USA..

[30] Nakazawa M, Mitjavila M, Debili N (1989). KU 812: A pluripotent human cell line with spontaneous erythroid terminal maturation. Blood.

[31] Younis MC (2021). Evaluation of deep learning approaches for identification of different corona-virus species and time series prediction. Comput. Med. Imaging Graph..

[32] Moses DA (2021). Deep learning applied to automatic disease detection using chest X-rays. J. Med. Imaging Radiat. Oncol..

[33] Li H, Weng J, Mao Y, Wang Y (2021). Adaptive dropout method based on biological principles. IEEE Trans. Neural
Networks Learn. Syst..

[34] Lin M, Wynne JF, Zhou B (2021). Artificial intelligence in tumor subregion analysis based on medical imaging: A review. J. Appl. Clin. Med. Phys..

[35] Li Y, Bidmeshki MM, Kang T, Nowak CM, Makris Y, Bleris L (2021). Provenance attestation of human cells using physical unclonable functions. bioRxiv..

[36] Shir, O. M., Raz, V., Dirks, R. W. & Bä́ck, T. Classification of cell fates with support vector machine learning. In *Lecture Notes in Computer Science (Including Subseries Lecture Notes in Artificial Intelligence and Lecture Notes in Bioinformatics)*. Vol 4447 LNCS, 258–269 ((Springer, 2007) 10.1007/978-3-540-71783-6_25.

[37] Lee KCM, Lau AKS, Tang AHL (2019). Multi-ATOM: Ultrahigh-throughput single-cell quantitative phase imaging with subcellular resolution. J. Biophotonics..

[38] Li Y, Mahjoubfar A, Chen CL, Niazi KR, Pei L, Jalali B (2019). Deep cytometry: Deep learning with real-time inference in cell sorting and flow cytometry. Sci. Rep..

