# Author Correction: Machine learning-based approaches for identifying human blood cells harboring CRISPR-mediated fetal chromatin domain ablations

**DOI:** 10.1038/s41598-024-54011-1

**Published:** 2024-02-12

**Authors:** Yi Li, Shadi Zaheri, Khai Nguyen, Li Liu, Fatemeh Hassanipour, Betty S. Pace, Leonidas Bleris

**Affiliations:** 1 Bioengineering Department, The University of Texas at Dallas, Richardson, TX USA; 2 Center for Systems Biology, The University of Texas at Dallas, Richardson, TX USA; 3 Department of Mechanical Engineering, The University of Texas at Dallas, Richardson, TX USA; 4 Department of Biological Sciences, University of Texas at Dallas, Richardson, TX USA; 5 Department of Pediatrics, Augusta University, Augusta, GA USA

Correction to: *Scientific Reports* 10.1038/s41598-022-05575-3, published online 27 January 2022

Betty S. Pace was omitted from the author list in the original version of this Article.

Consequently, in the Author Contributions section,

“Y.L., S.Z. and K.N. developed and performed the computational analysis. Y.L., K.N. and L.L. performed the experiments. Y.L., S.Z., K.N., F.H., and L.B. wrote the paper. L.B. supervised the project.”

now reads:

“Y.L., S.Z. and K.N. developed and performed the computational analysis. Y.L., K.N., B.S.P., and L.L. designed and performed the experiments. Y.L., S.Z., K.N., F.H., and L.B. wrote the paper. L.B. supervised the project.”

Also, in the Acknowledgements section,

“This work was funded by the US National Science Foundation (NSF) Grant 2029121, a Cecil H. and Ida Green Endowment, and the University of Texas at Dallas.”

now reads:

“This work was funded by the NIH grant HL069234 (B.S.P.), and US National Science Foundation (NSF) Grant 2029121, a Cecil H. and Ida Green Endowment, and the University of Texas at Dallas (L.B.).”

The original Article has been corrected.

