# Supplementary material for: Machine learning-based approaches for identifying human blood cells harboring CRISPR-mediated fetal chromatin domain ablations
Source: Sci Rep. 2022 Jan 27;12:1481. doi: 10.1038/s41598-022-05575-3 (PMC8795181; doi:10.1038/s41598-022-05575-3)
Supplement: Supplementary file 22 — Supplementary Information 22. [file 41598_2022_5575_MOESM22_ESM.docx]

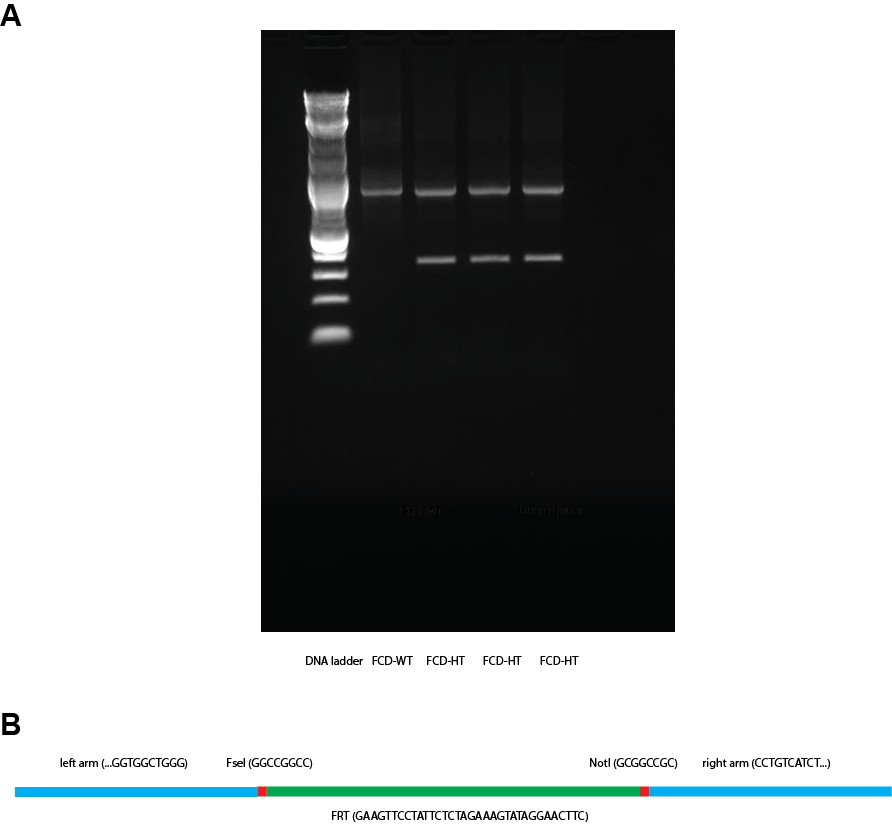


**Supplementary Figure 1. Genotyping of FCD-HT monoclonal stable cell. (A) DNA Gel electrophoresis results.** Genomic DNAs were harvested from FCD-WT and FCD-HT cells and the transcript containing the FCD motif or FRT site was PCR amplified and subsequently subjected to gel electrophoresis. The three independent replicates of stable FCD-HT cell line yielded two bands corresponding to both the wild type (806 bp) and FCD-knockout (341 bp) alleles, confirming its heterozygous status. DNA ladder: 1 kb Plus DNA Ladder (New England BioLabs, catalog number: N3200S). **(B)** Sanger sequencing result confirmed that the original FCD motif within human globin locus was removed in FCD-HT cell and replaced with a 50-bp FRT “scar” sequence.


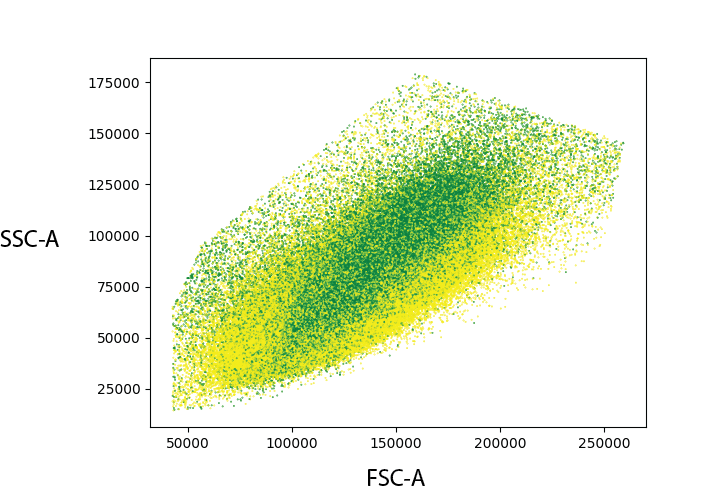


**Supplementary Figure 2. SSC-A vs. FSC-A plot for the training dataset (green: FCD-WT, yellow: FCD-HT).**


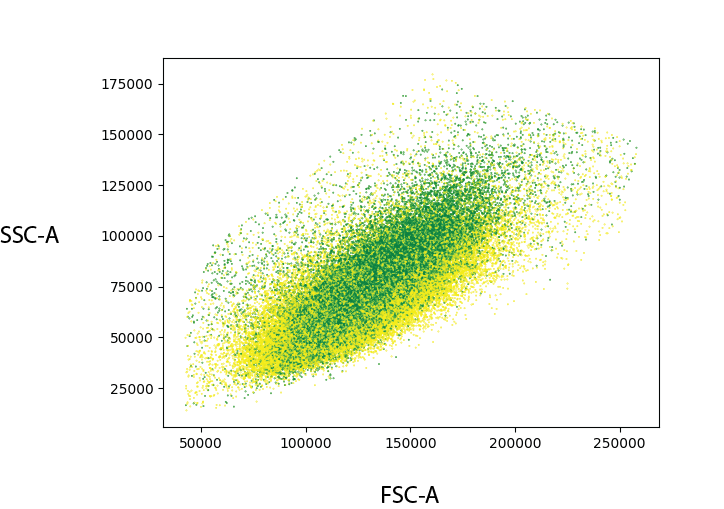


**Supplementary Figure 3. SSC-A vs. FSC-A plot for the testing dataset (green: FCD-WT, yellow: FCD-HT).**


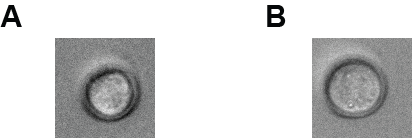


**Supplementary Figure 4. Representative DIC microscopy images for (A) FCD-WT and (B) FCD-HT cells.**


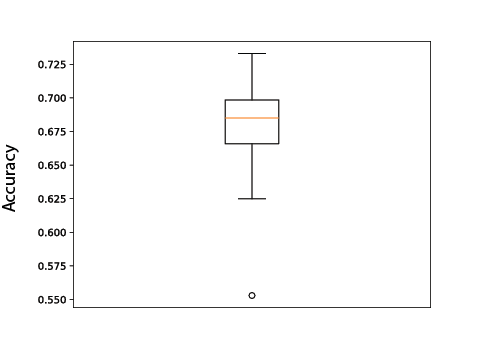


**Supplementary Figure 5. Box plot of accuracy values from the 10-fold cross-validation test using the T2D5 model.**


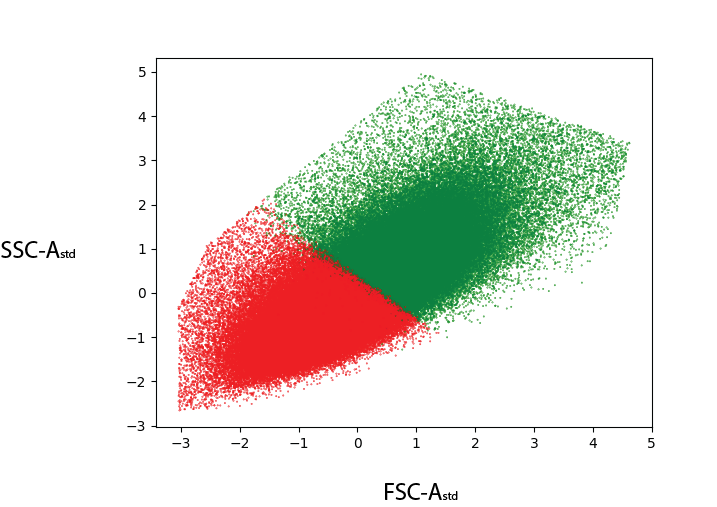


**Supplementary Figure 6. K-means clustering on standardized training dataset.** The model yielded poor performance when predicting FCD-HT cells.


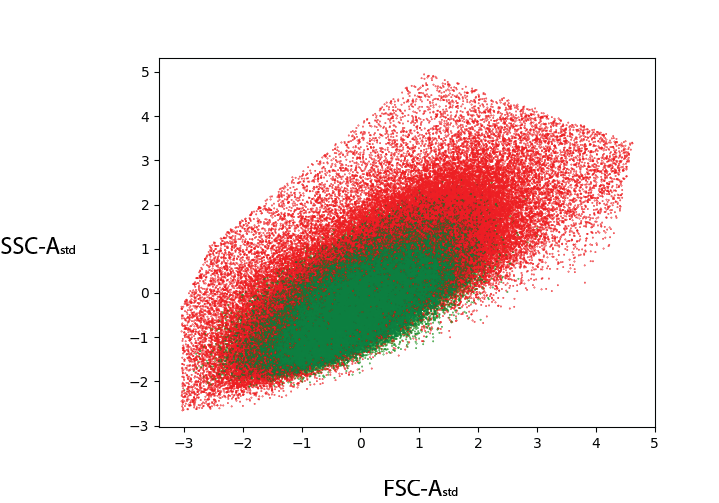


**Supplementary Figure 7. Gaussian mixture clustering on standardized training dataset.** The model yielded poor performance when predicting FCD-HT cells.


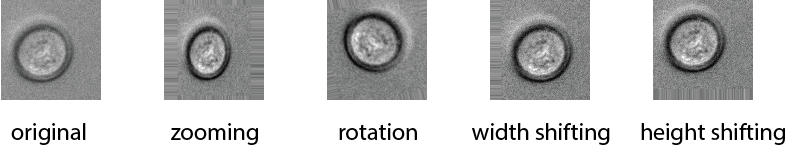


**Supplementary Figure 8. Data augmentation techniques applied to the training dataset.** The original images were subjected to zooming, rotation, width shifting and height shifting, resulting in a new training dataset with 26,604 images (FCD-WT: 12,897 images, FCD-HT: 13,707 images).


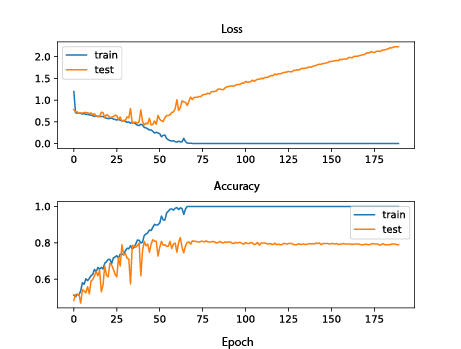


**Supplementary Figure 9. History of loss and accuracy values of training and testing datasets for the T2D5 model.** The discrepancy between the two datasets indicated that overfitting may have occurred.

**Supplementary Scripts. The cell morphology-based models (MLP_20_26.pkl, MLP_26_18.pkl, MLP_30_26.pkl, and T2D5.h5) and other main python scripts used in building and evaluating these predictive models in this study.**

**Supplementary Table 1. Primers used in this study.**

**Supplementary Table 2. Flow cytometry-based dataset.** In total, 192,772 FCD-WT cells (labeled as 0) and 185,544 FCD-HT cells (labeled as 1) were included in this study.

**Supplementary Table 3. The training dataset.** The training dataset contains 302,652 cells (label 0: 154,180 cells, and label 1: 148,472 cells).

**Supplementary Table 4. The testing dataset.** The testing dataset contains 75,664 cells (label 0: 38,592 cells, and label 1: 37,072 cells).

**Supplementary Table 5. The standardized training dataset.** The standardized training dataset contains 302,652 cells (label 0: 154,180 cells, and label 1: 148,472 cells).

**Supplementary Table 6. The standardized testing dataset.** The standardized testing dataset contains 75,664 cells (label 0: 38,592 cells, and label 1: 37,072 cells).

**Supplementary Table 7. The hyperparameters for five supervised learning algorithms (logistical regression, random forest, k-nearest neighbor, support-vector machine, and multilayer perceptron).**

**Supplementary Table 8. The mean and standard deviation values of 10-fold cross validation accuracy using the logistical regression algorithm.**

**Supplementary Table 9. The mean and standard deviation values of 10-fold cross validation accuracy using the random forest algorithm.**

**Supplementary Table 10. The mean and standard deviation values of 10-fold cross validation accuracy using the k-nearest neighbor algorithm.**

**Supplementary Table 11. The mean and standard deviation values of 10-fold cross validation accuracy using the SVM algorithm with linear kernel.**

**Supplementary Table 12. The mean and standard deviation values of 10-fold cross validation accuracy using the SVM algorithm with Gaussian kernel.**

**Supplementary Table 13. The mean and standard deviation values of 10-fold cross validation accuracy using the MLP algorithm.**

**Supplementary Table 14. The predictive performances of the 533 candidate models which passed both filtering conditions.**

**Supplementary Table 15. The architectural design and hyperparameters for seven deep learning-based convolutional neural networks used in this study.**

**Supplementary Table 16. The predictive performances of the seven deep learning-based convolutional neural networks used in this study.**

**Supplementary Table 17. The predictive performances of the T2D5 model using augmented training dataset.**

**Supplementary Table 18. The predictive performances of the T2D5 model using adjusted L2 regularization weights and dropout values.**

**Supplementary Table 19. Comparison between MLP and CNN methods used in this study.**

**Supplementary Table 20. The standardized testing dataset for HCT116 cells and their predicted labels based on MLP 20-26.** Label 0 (FCD-WT genotype) and label 1 (FCD-HT genotype)

**Supplementary Table 21. The standardized testing dataset for PUF3.1 HCT116 cells and their predicted labels based on MLP 20-26.** Label 0 (FCD-WT genotype) and label 1 (FCD-HT genotype)

**Supplementary Methods**

**General cloning protocols**

Q5 High-Fidelity 2X Master Mix (New England Biolabs) was used for all polymerase chain reactions (PCR) according to the manufacturer’s protocol. All oligonucleotides were ordered from Sigma-Aldrich and were listed in **Supplementary Table 1**. The plasmids were constructed using PCR amplification, restriction digest (all restriction enzymes were ordered from New England Biolabs), and ligation with T4 DNA ligase (New England Biolabs). Gel purification and PCR purification were performed with QIAquick Gel Extraction and PCR Purification kits (Qiagen). Transformations were performed using NEB 5-alpha electrocompetent *Escherichia Coli* (New England Biolabs). The minipreps were performed using QIAprep Spin Miniprep kit (Qiagen). The final plasmids were confirmed by both restriction enzyme digestions and direct Sanger sequencings.

**DNA Constructs**

**PCMV-SpCas9-U6-sgRNA-L:** The sgRNA sequence to target the upstream region of FCD was designed as 5’-TGGTGGTGGCTGGGGGGGC**TGG**-3’ (PAM sequence in bold and underlined). The U6-sgRNA-L sequence was PCR amplified from D12 (unpublished data) using primers P1 and P2, and subsequently P1 and P3, and subsequently cloned into D12 using KpnI and XbaI sites.

**PCMV-SpCas9-U6-sgRNA-R:** The sgRNA sequence to target the downstream region of FCD was designed as 5’-GCCCCTAGATCTGGCCAGG**TGG**-3’ (PAM sequence in bold and underlined). The U6-sgRNA-R sequence was PCR amplified from D12 (unpublished data) using primers P1 and P4, and subsequently P1 and P3, and subsequently cloned into D12 using KpnI and XbaI sites.

**Donor plasmid for generating the FCD-HT monoclonal stable cells:** The genomic DNA was isolated from wild type KU-812 cells using DNeasy Blood&Tissue Kit (Qiagen). The left arm was amplified with primers P5 and P6 using the KU-812 genomic DNA as the template, and then cloned into D25 vector (unpublished data) using ClaI and FseI sites. Next, the right arm was amplified with primers P7 and P8 using the KU812 genomic DNA as the template, and then cloned into the above plasmid using NotI and MscI sites. Next, the puromycin resistance gene transcript was amplified from D25 using primers P9 and P10. The mKate transcript was amplified from D12 (unpublished data) using primers P11 and P12. Subsequently, the puromycin resistance gene-T2A-mKate transcript was prepared using these two PCR products as template and primers P9 and P12, followed by subcloning into the above plasmid using AgeI and KpnI sites.

**FCD (Fetal Chromatin Domain) sequence**

GGCACCCTAGAACTCCCAAGAATATATGCCCTTTGTCTTCAGCTACCAGGGTGAGTAAGGAAGGACCATCAGGTGGGGGCAGGACTAGTCGTGTCTGAGCTCAGAGTCTCCTTGGGCAGGTCTTTCTGTGGCTACTGTGGGAGGATGGGGGTGTAGTTTCCAGGTCAATGGATTTATGTTCCTAGGACAATTATGGCTGCCTCTGCTGTGTCATGCAGGTCATCAGGAAAGTGGGGGAAAGCAAGCAGTCACGTGACTTGCCCAGCTCCCATGCAACTCAAAAGGTTGGTCTCACTTCCAGCGTGCACCCTCCCCCGCAACAGCACCGAATCTGTTTCCATGCAGTCAGTGAGCAAGGCTGAGAACTTGCCCCAGGCTACCAGCTGCGAAACCAAGTAGGGCTGTCCTACTTCCCTGCCAGTGGAGTCTGCACACCAAATTCATGTCCCCCCACCAACCCCCCCACTGCCCAGCCCCTAGATCTGGCCAGGTGGAGATTTTCT
